# Pancreatic Metastases from Clear Cell Renal Cell Carcinoma: Diagnostic Insights from Endoscopic Ultrasound-Guided Fine-Needle Biopsy

**DOI:** 10.3390/medicina62020239

**Published:** 2026-01-23

**Authors:** Alexandru Constantinescu, Ion Dina, Maria Nedelcu, Vlad Dumitru Băleanu, Vasile Florescu, Laura Enache, Octavian Andronic, Daniel Voiculescu, Ancuța Năstac

**Affiliations:** 1 Faculty of Medicine, Carol Davila University of Medicine and Pharmacy, 020021 Bucharest, Romaniadaniel.voiculescu@umfcd.ro (D.V.);; 2 Department of Gastroenterology, University Emergency Hospital Bucharest, 050098 Bucharest, Romania; 3 Department of Gastroenterology, Saint John’s Emergency Clinical Hospital, 042122 Bucharest, Romania; 4 Department of Surgery, University Emergency Hospital Bucharest, 050098 Bucharest, Romania; 5 Innovation and e-Health Center, Carol Davila University of Medicine and Pharmacy, 010194 Bucharest, Romania

**Keywords:** clear cell renal cell carcinoma, pancreatic metastases, endoscopic ultrasound, EUS-FNB

## Abstract

Clear cell renal cell carcinoma (ccRCC) is the most common type of kidney cancer, accounting for approximately 75–80% of all renal carcinomas, and is often diagnosed incidentally on abdominal imaging, such as abdominal ultrasound or CT scan. Among other types of renal cancer, ccRCC is recognized to be highly aggressive due to its metastatic potential, which leads to a poor prognosis and an increased mortality rate. The most common sites of ccRCC metastasis are the lung, lymph nodes, bone, liver, and adrenal glands. Clear cell RCC is the most frequent primary tumor associated with secondary pancreatic involvement, while overall, pancreatic metastases represent only 2–5% of all malignant pancreatic lesions. These metastases often occur many years after nephrectomy and may present as solitary or oligometastatic disease, frequently displaying a paradoxically favorable prognosis compared with other metastatic sites. The present narrative review we conducted emerged from presentations of ccRCC with pancreatic distant metastases, potentially labeled as primary pancreatic tumors on imaging studies, mimicking pancreatic neuroendocrine tumors due to the hypervascular nature of ccRCC. Four patients were investigated in our clinic for suspicious pancreatic lesions identified on CT imaging, involving both the head and body of the pancreas. The definitive diagnosis was established by performing endoscopic ultrasound-guided fine-needle aspiration (EUS-FNA) or fine-needle biopsy (FNB) and histopathological analysis of the collected tissue samples. Endoscopic ultrasound-guided fine-needle biopsy (EUS-FNB) has emerged as a pivotal tool for obtaining tissue diagnosis, particularly when cross-sectional imaging is inconclusive. Through a synthesis of clinical data and literature, this article underscores the essential diagnostic role of EUS-guided tissue acquisition and its impact on therapeutic decision-making.

## 1. Introduction

Between 1975 and 2025, more than 6500 publications with titles referencing “metastasis to the pancreas” were indexed in the Web of Science (WOS) database (by Clarivate Analytics), consisting primarily of original articles along with fewer review articles and case reports [1,2,3,4,5,6,7,8,9,10]. If we refined our search, considering only pancreatic metastases from renal clear cell carcinoma, almost 200 papers have been published in this period. Within this body of literature, case reports highlighted atypical presentations, emphasizing the heterogeneity of clinical patterns, especially the character of a late-relapsing disease [11,12,13,14,15,16,17,18]. This narrative review emerged from the need to integrate focal pancreatic masses within the broader context of secondary lesions, particularly in patients with a history of renal cancer, even when the primary tumor was diagnosed decades earlier. Recognizing this association is crucial, as pancreatic metastases from renal clear cell carcinoma may appear long after nephrectomy and can mimic primary pancreatic neoplasms, making timely identification and accurate differential diagnosis essential.

Renal cell carcinoma (RCC) accounts for 2–3% of all adult malignancies, with clear cell renal cell carcinoma (ccRCC) representing its predominant histological subtype, responsible for approximately 75–80% of cases [19]. Despite advances in oncologic therapies, ccRCC remains an aggressive malignancy with an unpredictable metastatic pattern. The management of metastatic renal cell carcinoma (mRCC) has undergone a significant paradigm shift toward multimodal strategies involving targeted therapies and novel immunotherapy with immune checkpoint inhibitors; however, the general 5-year survival rates for mRCC historically remain low, ranging between 0% and 20% [20]. Late recurrences, sometimes appearing more than a decade after nephrectomy, are well documented, illustrating the long-evolving natural history of this tumor type [21,22,23]. This delayed manifestation with the development of a secondary pancreatic solid mass risks being misinterpreted as a primary pancreatic tumor, posing challenges for therapeutic management and follow-up.

The pancreas is a rare site for metastatic disease overall, but it represents the most frequent location for metastases originating from RCC, particularly ccRCC [24,25,26,27,28]. Pancreatic metastases demonstrate unique biological behavior: they are often hypervascular, may occur metachronously after prolonged disease-free intervals, and remarkably, are associated with a significantly better prognosis compared with other forms of metastatic RCC, with reported 5-year survival rates ranging from 60 to 80% [26,27,29,30].

The diagnosis may be challenging because pancreatic metastases from ccRCC can mimic other hypervascular pancreatic neoplasms, especially neuroendocrine tumors. While contrast-enhanced CT and MRI are useful, EUS-guided fine-needle aspiration or biopsy (EUS-FNA/FNB) remains the gold standard for obtaining a definitive diagnosis, particularly when imaging findings are equivocal [31,32,33]. Accurate identification of metastatic ccRCC is crucial, as this aggressive malignancy requires tailored therapeutic approaches.

Our study integrates current clinical evidence with four clinically illustrative cases evaluated in our tertiary center, all diagnosed using EUS-FNA/FNB. Each case highlights specific diagnostic and procedural challenges, including hypervascularity, lesion heterogeneity, and the occasional limitations of immunohistochemistry in resource-constrained settings. Moreover, it gave insight into the rare co-occurrence of clear cell renal carcinoma and colonic adenocarcinoma through a review of the literature.

The aim was to provide practical diagnostic insights into pancreatic metastases from ccRCC and to highlight the essential role of EUS-guided tissue acquisition in establishing a definitive diagnosis and guiding management.

Early and accurate differentiation between primary pancreatic tumors and metastatic ccRCC is critical, as management strategies and prognosis differ substantially.

## 2. Materials and Methods

Between 2018 and 2021, a total of 1254 patients were evaluated in our tertiary Endoscopy Department for pancreatic lesions identified on CT imaging. Among them, four patients had a known history of ccRCC and presented with pancreatic lesions requiring EUS-guided tissue sampling.

Subsequent diagnostic evaluation with endoscopic ultrasound-guided fine-needle aspiration or biopsy (EUS-FNA/B) confirmed pancreatic metastases from ccRCC, which constituted the inclusion criteria for this study.

All procedures were performed by two endoscopists, while histopathological examinations were carried out by a single pathologist.

All EUS procedures were performed using a linear echoendoscope. Tissue acquisition was performed using 22-gauge FNA or FNB needles, Boston Scientific™ Expect and Acquire (Boston Scientific, Marlborough, MA, USA). Rapid on-site evaluation (ROSE) was used for the first two cases.

Morphological and immunohistochemical studies were performed according to standard national protocols. However, the assessment was limited by the absence of certain immunohistochemical markers required for accurate assessment.

Written informed consent was obtained from all patients at the time of the procedure, while retrospective ethical approval was subsequently granted by the hospital’s ethics committee.

This work is structured as a narrative review illustrated by clinical cases evaluated in our department. To better contextualize Case 4, a rare presentation of late-recurrent ccRCC synchronous with colonic adenocarcinoma, we performed a targeted literature search in PubMed and Web of Science (2000–2025). This supplementary review aims to explore the rare association between these two primary malignancies, which can complicate both diagnostic pathways and surgical planning. For this review, we searched the databases for terms related to colonic involvement (“colon*”, “colorectal*”, “sigmoid*”), renal involvement (“kidney”, “renal”), and neoplasia (“adenocarcinom*”, “neoplasm*”, “malign*”, “clear cell”, “carcinom*”), in the 2000–2025 interval, in the English language. The articles were selected initially based on title and abstract, and then were further filtered for the specific malignant subtypes through full-text evaluation.

## 3. Results

### Clinical Illustrations

In the current study, endoscopic ultrasound was performed for pancreatic metastases caused by ccRCC in 4 patients. Among them, the gender ratio was 2/2, with an average age of 69 years. They were located both cephalically and caudally, with dimensions between 22 and 68 mm, different Doppler signal intensities, and hard or mixed in consistency at elastography.

In our series, the diagnosis of pancreatic metastasis from ccRCC was established based on a combination of:

1. A documented clinical history of primary renal cell carcinoma;
2. Endoscopic ultrasound findings, typically hypoechoic, well-defined lesions often exhibiting hypervascularity on Doppler;
3. Histopathological and immunohistochemical (IHC) confirmation via EUS-FNB/FNA, characterized by clear cell morphology and a positive IHC profile for PAX8, CAIX, CD10, and vimentin.

The primary risk of misclassification involves primary pancreatic neuroendocrine tumors, which are also hypervascular on imaging. To mitigate this risk, negative staining for neuroendocrine markers such as chromogranin and synaptophysin was essential to confirm the metastatic nature of the lesions.

**Figure 1:**
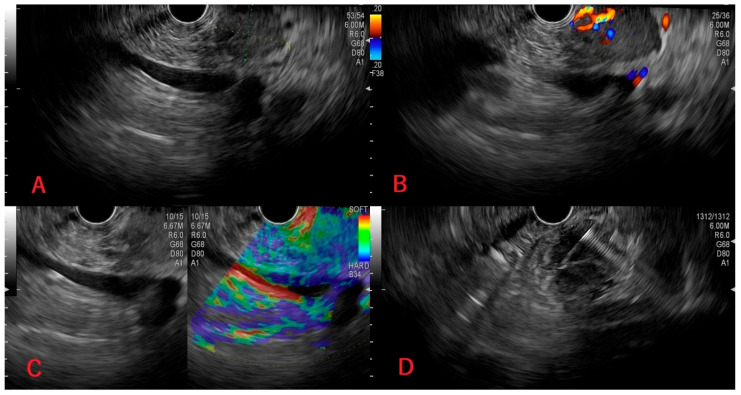
Case 1: Hypoechoic pancreatic tumor measuring 21/16 mm (**A**), intense Doppler signal (**B**), hard on elastography (**C**); tissue acquisition through EUS-FNB (**D**).

**Figure 2:**
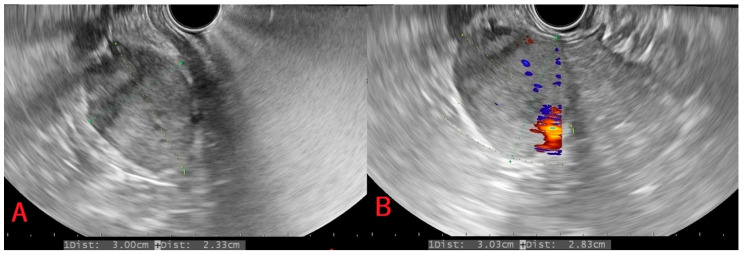
Case 2: Head of the pancreas tumor hypoechoic on ultrasound (**A**) with normal intensity Doppler signal (**B**).

**Figure 3:**
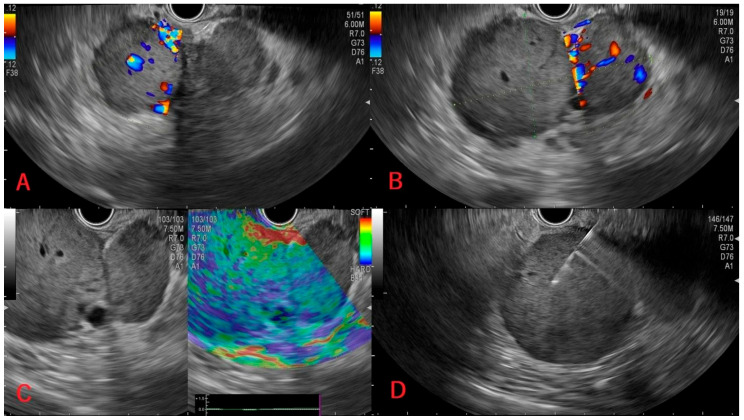
Case 3: Ultrasonography with an intense Doppler signal in both lobes of the pancreatic lesions (**A**,**B**), hard consistency of the pancreatic tumor (**C**), and tissue sampling through EUS (**D**).

**Figure 4:**
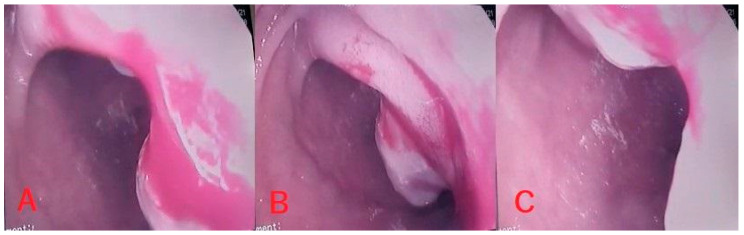
Case 4: Endoscopic aspect of protrusive duodenal lesion (**A**); hemostasis with Adrenalin 1:10,000 injection (**B**) and bipolar electrocoagulation (**C**).

Following biopsy, samples were sent for histopathological examination, and the presence of pancreatic metastases from ccRCC was revealed in all four patients. Table 1 shows a summary of the cases. Figure 1, Figure 2, Figure 3 and Figure 4 shows available images from the cases.

In the following Figure 1, Figure 2, Figure 3 and Figure 4, there are shown images of the four cases of pancreatic metastases of clear cell renal cell carcinoma.

## 4. Discussion

### 4.1. Biological Mechanisms of Pancreatic Metastasis in ccRCC

From a molecular perspective, ccRCC metastasis is largely driven by inactivation of the von Hippel–Lindau (VHL) gene, which is a tumor suppressor gene that regulates cell division, apoptosis, and cell differentiation and is found in more than 90% of ccRCC [34,35,36,37]. The result is stabilization of hypoxia-inducible factors and subsequent upregulation of pro-angiogenic pathways such as VEGF and PDGF [38,39,40,41]. These molecular alterations promote angiogenesis, tumor proliferation, and metastatic dissemination. In addition, tumor–immune interactions—including PD-L1 expression and recruitment of immunosuppressive cell populations—further contribute to metastatic potential and disease progression. The pancreas provides a permissive microenvironment for metastatic implantation, characterized by rich vascularization, favorable stromal interactions, and an immune microenvironment promoting tumor persistence, consistent with the “seed and soil” hypothesis [42,43,44,45].

### 4.2. Clinical Presentation and Prognostic Features

Clear cell renal carcinoma is a major cause of morbidity and mortality among patients with kidney cancer, representing more than three-quarters of all cases [46]. Metastatic spread to multiple organs, including the lungs, lymph nodes, bone, liver, and adrenal glands, and only exceptionally to the pancreas, constitutes a major challenge in clinical practice [47,48,49]. Clear cell renal carcinoma is the most frequent primary tumor that metastasizes to the pancreas, despite the pancreas being an exceptionally rare site of metastatic spread [50,51,52]. A few sporadic cases of urogenital, breast, or lung tumors have been reported over time to disseminate to the pancreas [3,53,54,55]. In contrast to ccRCC, ovarian and colorectal cancers are among the rarest malignancies to spread to the pancreas. In such cases, immunohistochemical analysis plays a crucial role in distinguishing primary pancreatic tumors from metastatic lesions [56,57].

The majority of patients may remain asymptomatic for extended periods of time, with lesions detected incidentally during surveillance imaging or workup for nonspecific abdominal complaints [30,46]. When symptoms do occur, patients most commonly present with abdominal pain, jaundice, pancreatitis-like presentations, or gastrointestinal bleeding. A paradoxical survival benefit has been consistently observed in patients with pancreatic metastases compared with those with metastases at non-pancreatic sites [58]. Other reports also indicate that less aggressive tumor clones preferentially metastasize to the pancreas [27]. The phenomenon of isolated pancreatic involvement has been consistently described in retrospective series and case reports, suggesting a biological predilection rather than a random distribution of metastatic disease [27,28].

Our case study highlighted a heterogeneous behavior of this tumor type: whereas two patients deteriorated quickly as a result of systemic disease progression, the first and last patients achieved extended survival after combined surgical and oncological treatment. This heterogeneity suggested that pancreatic metastases may represent a clinically relevant subset of metastatic ccRCC, potentially explained by the “seed and soil” hypothesis, where the pancreatic microenvironment provides a niche more compatible with indolent tumor clones, due to rich vascularization and immune environment. The prognosis of ccRCC patients with pancreatic metastases remains difficult to predict, given the tumor’s aggressive metastatic nature and associated poor overall outcomes.

A defining characteristic of ccRCC is its propensity for late-onset metastases, which may appear many years—even decades—after radical surgery [29,59]. Although nephrectomy is the standard treatment for localized RCC, metastatic progression still occurs in up to 30% of patients following complete surgical resection, usually in the pancreas, underscoring the unpredictable and often protracted natural history of the disease [11,12,13,60,61,62,63,64]. This was evident in the fourth case we presented, which suffered re-occurrence after 20 years from the initial diagnosis.

### 4.3. The Co-Occurrence of Renal Clear Cell Carcinoma and Colonic Adenocarcinoma

Our review identified 9 articles (case reports or retrospective studies) that gave insight specifically about the co-occurrence of clear cell renal cell carcinoma and colonic adenocarcinoma (Table 2). A few other studies were identified that were published before 2000, highlighting that this co-occurrence has been well documented for an extended period of time. However, this mini-review is useful for some clinical considerations. Firstly, most of the cases in this review did not have metastases at presentation and had a relatively asymptomatic renal neoplasia that was discovered due to symptoms caused by the colonic neoplasia or other simultaneous neoplasias (however, one case presented for symptoms caused by the renal cell carcinoma, i.e., lymphatic node enlargement). Despite that, in our case, the patient presented with a late recurrence of renal cell carcinoma (after more than 20 years), which manifested with upper gastrointestinal bleeding through a pancreatic metastasis infiltrating the duodenum and led to the diagnosis of an asymptomatic colonic adenocarcinoma.

We did not evaluate in our clinic for the presence of predisposing syndromes; they were objectified intermittently in the literature (Table 2). Even if a clear connection with a particular syndrome, such as von Hippel–Lindau or hereditary nonpolyposis colorectal cancer, might not always be established, a rise in secondary malignancy risk is clearly documented for patients with renal carcinoma, although in some studies, the clear cell subtype is less involved in such predispositions [65].

**Table 2:** Summary of literature cases reporting pancreas metastases from ccRCC.

Author, Year	Type	Age/Sex	Presentation	Clinical Features	Metastases	Outcome	Commentaries of Retrospective Analyses
Plangsiri, 2025 [66]	Case series	Patient 1: 65/F	Hematochezia for 7 months	Sigmoid adenocarcinoma and renal clear cell carcinoma—no underlying predisposing factor mentioned	Not identified	Good postoperative outcome, unclear follow up duration	
		Patient 2: 73/F	Severe abdominal pain exacerbated by movement	Sigmoid adenocarcinoma and renal clear cell carcinoma—no underlying predisposing factor mentioned	Not identified	Good postoperative outcome, unclear follow up duration	
Naik, 2020 [67]	Case report	65/M	Abdominal pain, dorsalgia	Adenocarcinoma of the ascending colon and clear cell carcinoma of the kidney—no underlying predisposing factor mentioned	Not identified	Good postoperative outcome, 6 months follow-up	
Dafashy, 2016 [68]	Case series	36/F	Left supraclavicular neck mass	Clear cell renal cell carcinoma and colonic adenocarcinoma in the context of hereditary nonpolyposis colorectal cancer, without any known family history	Renal cell carcinoma (lymph nodes).	Renal recurrence after one month with extending lymphatic involvement. The patient died approximately one year after diagnosis.	
Jin Heo, 2016 [69]	Case report	74/M	Gait disturbance and tingling sensation of both legs	Colorectal adenocarcinoma, renal clear cell carcinoma and hemangioblastomas in von Hippel–Lindau disease	Metastases of hemangio-blastoma (brain), colonic adeno-carcinoma (liver), renal cell carcinoma (aortocaval lymph node)	The patient was stable at the writing of the paper (approximately one year later)	
Zinnamosca, 2013 [70]	Case report	64/M	Intermittent abdominal pain, loss of appetite and constipation	Colon adenocarcinoma, renal clear cell carcinoma and adrenal pheochromo-cytoma in the context of Von Hippel-Landau disease.	Not identified at presentation; renal clear cell metastases appeared after 9 years of monitoring: lung, lymph nodes, mediastinum, liver.	Death 11 years after the initial presentation	
Steinhagen, 2013 [71]	Retrospective study						A study on 24,642 patients with colorectal cancer and 7366 patients with cell renal carcinoma (64% clear cell): synchronous occurrence in 0.73% of colonic neoplasias and 2.4% of renal neoplasias. Median age at diagnosis: 64 (28–80); 76% males; most tumors were in early stages; only one patient had pancreatic involvement from the renal malignancy; 42% of patients had at least one more neoplasia. The patients could not be associated with the Lynch syndrome.
Kozokic, 2011 [72]	Case report	81/M	Melena, intermittent abdominal pain and loss of appetite	Adenocarcinoma of the sigmoid colon and clear cell carcinoma of the kidney—no underlying predisposing factor mentioned	Not identified.	Good recovery after surgery; unclear data regarding follow-up	
Thompson, 2006 [63]	Retrospective study						2722 patients with renal cell carcinoma: clear cell carcinoma was less associated with colon cancer and second malignancy than other types of renal cancer
Capra, 2003 [73]	Only abstract, case series	Three patients	Patients were initially investigated for the colorectal neoplasia (the renal malignancy was asymptomatic)				

### 4.4. Tumor Identification

In the cases we presented, the diagnosis of ccRCC was based not only on the expression of immunohistochemical markers (such as CD10, AE1/AE3, vimentin), but on a comprehensive approach that integrated the clinical context, histopathological morphology, and the exclusion of other differential diagnoses. For example, the negative staining for chromogranin, synaptophysin, CK7, and CK20 effectively ruled out neuroendocrine and primary pancreatic tumors, further supporting the diagnosis of metastatic ccRCC. Furthermore, a thorough history could be needed in order to identify earlier diagnoses of ccRCC, which could have happened more than 10 years prior and might not immediately come to mind for the patient.

Regarding diagnostic tumor markers, clear cell renal carcinoma currently lacks any specific serological indicators. Nevertheless, occasional elevations of CA125 have been reported in tumors of the upper urinary tract, especially in clear cell tumors arising from the upper ureter and renal pelvis [74,75]. Its elevation, when present, is considered nonspecific and may reflect peritoneal irritation, inflammatory response, or ectopic production by urothelial tumor cells [76]. Rare reports have documented serum CA125 elevation, particularly in advanced or invasive disease, suggesting a potential—but unvalidated—role as an indicator of tumor burden or disease progression. CA125 may serve as a useful marker during follow-up to assess complete tumor remission or to monitor for recurrences [74].

A retrospective study conducted on 15 patients concluded that the pattern of enhancement and imaging characteristics of the lesions help differentiate the metastatic disease from primary pancreatic tumors and neuroendocrine tumors (functional or nonfunctional) [77]. Accurate diagnosis is critical, as therapeutic strategies differ substantially between primary pancreatic malignancies and metastatic ccRCC. A key feature of ccRCC metastases is their hypervascularity, which has both diagnostic and therapeutic implications. On imaging, this vascular pattern can mimic primary pancreatic neuroendocrine tumors, posing a challenge for differential diagnosis [78,79]. From a procedural standpoint, the pronounced hypervascularity predisposes patients to complications such as hematoma formation following fine-needle aspiration or biopsy, as illustrated in one of the presented cases. This risk underscores the importance of careful procedural planning and close post-procedural monitoring, particularly in anticoagulated or comorbid patients. However, two of the cases we presented did not have hypervascularity. Those heterogeneous manifestations must argue for careful clinical evaluation of each case.

It is worth noting that in healthcare systems where access to full IHC panels may be limited, such as in Romania, due to cost constraints or local availability, the diagnostic pathway for pancreatic metastases from ccRCC often follows a stepwise approach. Initial testing is typically focused on excluding more common differentials such as lymphoma or neuroendocrine tumors. If these diagnostic approaches yield negative results, a presumptive diagnosis of ccRCC can be supported by morphological evaluation with standard Hematoxylin–Eosin staining, in conjunction with the clinical history of a synchronous or metachronous renal tumor. In such situations, the integration of pathology with clinical and imaging findings can strongly suggest ccRCC, although confirmation via immunohistochemistry remains the gold standard whenever possible.

Eleven patients with ccRCC and pancreatic metastases were examined in a clinical study, with a focus on immune cell infiltration in primary tumors and pancreatic metastases. Immunohistochemical analysis revealed a reduced density of immune cells, including CD45+ immune cells, CD8+ cytotoxic T cells, FOXP3+ regulatory T cells, and CD163+ macrophages, in pancreatic metastases compared to primary tumors and RCC without pancreatic involvement. The low presence of CD8+ T cells, which are associated with anti-tumor activity, and the reduced levels of FOXP3+ T regulatory cells, known for their immunosuppressive role, present a distinct immune landscape in pancreatic metastases [80].

### 4.5. Diagnostic Efficacy of EUS-FNA/FNB

While pancreatic metastases from ccRCC often appear as hyperenhancing lesions on CT/MRI, mimicking neuroendocrine tumors, imaging remains non-diagnostic in atypical cases [77]. In these instances, EUS-FNA/FNB is the gold standard, offering a diagnostic accuracy of 86% [31,81,82,83,84,85,86].

The technical superiority of EUS lies in its real-time visualization and ability to characterize vascularity. Specifically, EUS-FNB facilitates the preservation of tissue architecture, which is essential for immunohistochemical staining to differentiate metastatic ccRCC from primary pancreatic malignancies [31,32]. Large-scale data (*n* = 1161 procedures) confirms that these metastases typically present as hypoechoic, well-defined lesions (97.56%). Notably, EUS can detect diffuse pancreatic heterogeneity in symptomatic patients even when conventional contrast-enhanced CT appears normal, highlighting its role when clinical suspicion remains high despite negative cross-sectional imaging [33,84,87].

### 4.6. Clinical Management and the Era of Immunotherapy

Management of pancreatic metastases from ccRCC should be individualized according to disease extent, patient comorbidities, and tumor biology. Surgical resection remains the gold standard for selected patients with isolated or oligometastatic disease, offering 5-year survival rates of 60–80%, significantly higher than those reported for pancreatic adenocarcinoma or ccRCC metastases to other organs [26,47,88,89]. For patients with unresectable or disseminated disease, systemic approaches, including VEGF inhibitors, tyrosine kinase inhibitors, and immune checkpoint inhibitors, have improved outcomes, while optimal integration with surgery requires a multidisciplinary strategy [79,90]. Multicentric analyses have shown that patients receiving local pancreatic treatments combined with targeted therapy exhibit superior survival, supporting the potential benefit of an aggressive yet personalized therapeutic approach [91,92,93,94]. In cases with limited metastatic burden, focal treatments can achieve durable local control and delay systemic therapy initiation [95,96,97,98,99]. Within this evolving landscape, endoscopic ultrasound-guided radiofrequency ablation (EUS-RFA) has emerged as a minimally invasive alternative to surgery, demonstrating local control rates of 84% at 6 months and 73% at 12 months in the largest prospective study to date [100]. Subsequent series have confirmed its feasibility and high technical success, particularly for lesions under 15 mm, underscoring its suitability for small, localized metastases or patients unfit for major surgery [101,102]. Importantly, the integration of EUS-FNA/FNB findings into therapeutic decision-making is crucial in the immunotherapy era. The combined use of direct smear cytology and the cell block technique allows for accurate morphological and immunocytochemical characterization, enabling precise differentiation between metastatic ccRCC and primary pancreatic neoplasms and thus guiding appropriate treatment pathways [103].

### 4.7. Limitations

Our study has several limitations that should be considered when interpreting the results. First, the clinical series is limited to four cases collected retrospectively, which inherently restricts the ability to draw broad statistical conclusions. Furthermore, real-world diagnostic constraints resulted in incomplete IHC panels for some patients, although definitive diagnoses were supported by integrating clinical history with morphological and available molecular data. The follow-up period was also relatively short, potentially underestimating long-term survival outcomes in this characteristically indolent disease. Finally, while we provide a detailed analysis of the co-occurrence of ccRCC and colonic adenocarcinoma, the narrative nature of this review may not capture the full breadth of emerging data as a systematic review would. Despite these constraints, this series offers a valuable regional perspective from Eastern Europe and highlights the pragmatic utility of EUS in diverse clinical environments.

## 5. Conclusions

Pancreatic metastases from ccRCC represent a unique clinical entity characterized by late recurrence, often manifesting decades after the primary nephrectomy, and a paradoxically favorable prognosis compared to other metastatic sites. This work reinforces that EUS-FNA/FNB has evolved from an elective diagnostic tool into a cornerstone of multidisciplinary management. Its high sensitivity and specificity are essential for resolving the diagnostic ambiguity created by the hypervascular nature of these lesions, which frequently mimic primary pancreatic neuroendocrine tumors on conventional imaging. Beyond simple identification, the transition to EUS-FNB provides the architectural and cellular detail necessary for modern precision medicine. In the era of immunotherapy and targeted TKI protocols, the ability to obtain high-quality core biopsies for molecular and immune-landscape profiling is critical for patient stratification. Furthermore, emerging minimally invasive techniques like EUS-guided radiofrequency ablation represent a promising frontier for achieving local disease control in patients unfit for major surgery. We conclude that clinical pathways for ccRCC survivors must prioritize early EUS intervention for even subtle pancreatic findings. Such a proactive approach optimizes the window for both curative surgical resection and advanced systemic interventions, ultimately capitalizing on the indolent nature of these metastases to maximize long-term patient survival.

## Figures and Tables

**Table 1:** Summary of the cases.

Case	Age/Sex	Presentation & Relevant History	Lesion Location & Size	EUS Appearance	Key IHC Findings Reported	Complications	Management & Follow-Up
1	65/M	Incidental lesion on CT (admission for cholecystectomy); renal tumor diagnosed during the same presentation; cardiovascular comorbidities.	Cephalic, 22/16 mm.	Hypoechoic, well-demarcated, relatively homogeneous; accentuated intratumoral vascular flow; hard consistency.	Negative: chromogranin, synaptophysin, CK7, CK20. Positive: cytokeratin AE1/AE3, CD10, vimentin, PAX8 and CAIX.	No significant short-term complication.	CT→EUS with FNA→Single-stage nephrectomy + cephalic duodenopancreatectomy; oncologic treatment; favorable outcomes at 3-year follow-up.
2	72/M	Known left renal tumor and pulmonary metastases—a caudal pancreatic lesion apparent on CT; cardiovascular comorbidities, cholecystectomy	Cephalic, 30 × 28 mm.	Hypoechoic; normal Doppler signal; hard.	Negative: chromogranin, synaptophysin, CK7, CK20, p63, HSA. Positive: AE1/AE3, PAX8, CAIX.	Perilesional fluid outline ~3 cm, suggestive of de novo hematoma. It did not increase in size and Hb remained normal.	CT→EUS with FNA→Oncologic treatment only (advanced stage). Unknown follow-up data.
3	61/F	Referred for painless jaundice and mild pruritus. CT: small renal tumor, cephalo-pancreatic lesion, lung/liver metastases. No significant medical history.	Uncinate process; bilobed; 68 × 41 mm.	Hypoechoic, rich Doppler signal; predominantly hard consistency.	Negative: chromogranin, synaptophysin, CK7, CD10, AE1/AE3. Positive: Ki-67 (5–10%), PAX8 CAIX.	No significant short-term complication.	CT→EUS with FNA→Oncologic treatment only (advanced stage); cardiac arrest during hospitalization.
4	78/F	Recurrent UGIB which revealed a pancreatic lesion bulging and infiltrating in the second part of the duodenum and an anatomic vascular variant with a replaced right hepatic artery arising from the superior mesenteric artery; history of ccRCC (more than 20 years prior, grade 2, surgically managed); simultaneously diagnosed sigmoid adenocarcinoma (due to the appearance of wall thickening on CT).	Pancreatic lesion bulging and infiltrating in the second part of the duodenum.	Irregular, round, intense Doppler vascular signal, hard consistency.	Limited IHC evaluation, but PAX8 positive	Recurrent hemorrhagic episodes.	Endoscopic hemostasis with initial inconclusive biopsy→CT→EUS with FNA→ left colectomy and cephalic duodenopancreatectomy; favorable outcome maintained at 2-year follow-up.

## Data Availability

The data presented in this study are available on request from the corresponding author.

## Abbreviations

The following abbreviations are used in this manuscript:

RCC	renal cell carcinoma
ccRCC	clear cell renal carcinoma
mRCC	metastatic renal cell carcinoma
EUS	endoscopic ultrasound
ROSE	rapid on-site evaluation
FNA	fine-needle aspiration
FNB	fine-needle biopsy
RFA	radio-frequency ablation
TT	targeted therapies
